# Climatic effects on aflatoxin contamination of maize

**DOI:** 10.1016/j.toxrep.2024.101711

**Published:** 2024-08-15

**Authors:** Queenta Ngum Nji, Olubukola Oluranti Babalola, Mulunda Mwanza

**Affiliations:** a Food Security and Safety Focus Area, Faculty of Natural and Agricultural Sciences, North-West University, Private Bag X2046, Mmabatho 2735, South Africa; b Department of Animal Health, Faculty of Natural and Agricultural Sciences, North-West University, Private Bag X2046, Mmabatho 2735, South Africa

**Keywords:** Aflatoxins, Food safety, HPLC, Maize, South Africa

## Abstract

Aflatoxins are frequent contaminants of maize especially in the face of climate change with deleterious health and socio-economic impacts. South Africa is ranked 9th maize exporter globally; hence, insights need to be gained in terms of the maize value chain in South Africa with respect to aflatoxin contamination to evaluate consumers’ exposure. High-performance liquid chromatography (HPLC) technique was used in this study to quantify aflatoxins in South African commercial maize. One thousand and twenty-eight (1028) maize samples were collected across six distinct agro-climatic regions over five harvest seasons (2017 – 2021). A total of 205 samples (19.94 %) were found to be contaminated with aflatoxins, with mean total aflatoxin concentration of 64.17 ppb amongst the contaminated samples, which is above the SA regulatory limit of 20 ppb for animal consumption. The year 2018 recorded the highest mean total aflatoxin value while North-West agro-climatic region had the highest mean total aflatoxin value. Drastic reduction in average rainfall significantly influence aflatoxin contamination of South African maize.

## Highlights

• Continuous monitoring of the growth of toxigenic fungi and aflatoxin contamination of maize in the face of climate change is essential for consumers’ safety and policy makers.
• Chromatographic techniques remain the most reliable methods for aflatoxin detection and quantification in food.
• Drastic reduction in rainfall increases aflatoxin contamination in maize.

## Graphical Abstract

**Figure:**
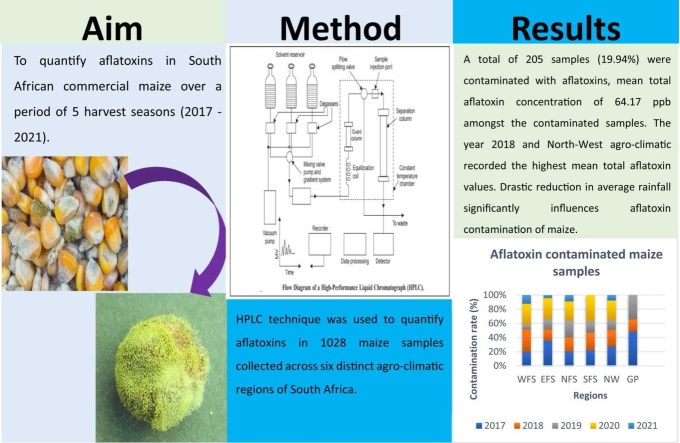

## 1 Introduction

Mycotoxins are toxic chemicals produced by filamentous fungi which are commonly found in agricultural produce such as peanuts, cereals, especially maize. High rates of aflatoxin contamination of maize between 70 % and 100 % have been reported in Africa [1], [2], [3], [4], [5], [6], [7] and could be attributed to Africans total dependent on climatic variables for agricultural production [8]. Climatic indicators such as temperature, rainfall and carbon dioxide levels have increased lately with increased in pre-harvest aflatoxin risk associated with crops [9]. South Africa is experiencing frequent droughts, drastic reduction in rainfall and rising temperatures which are estimated to rise at a pace double the global rate alongside sporadic rainfall and for every 2˚C rise in temperature, there is a substantial pre-harvest aflatoxin risk associated with maize [9], [10], [11], [12], [13], [14]. Maize is a staple diet for more than 200 million people in the developing world [15]. South Africa a leading exporter of maize on the continent and is ranked ninth globally [16]. It is estimated that the demand for maize will increase by nearly 300 million tons by 2030 [17]. Therefore, maize cultivated in South Africa under the aforementioned climatic scenarios might be at pre-harvest aflatoxin risk contamination. Hence, the aim of this study is to quantify aflatoxins in South African commercial maize over a period of 5 years.

## 2 Materials and methods

### 2.1 Sampling sites

**Fig. 1:**
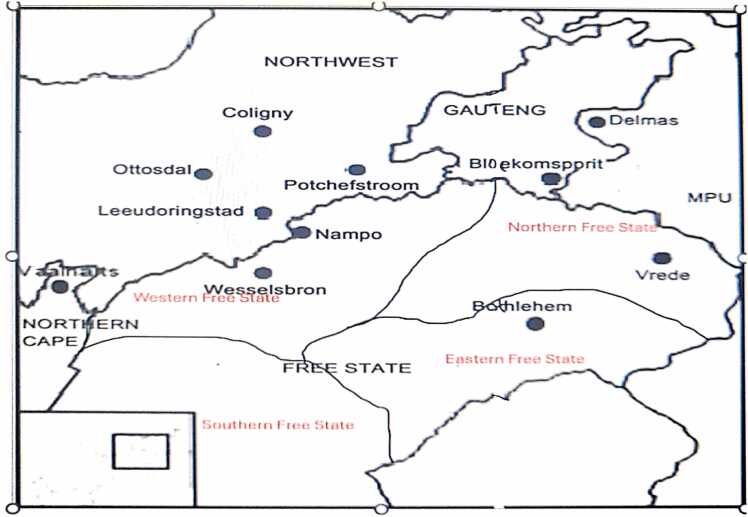
Map showing areas in south Africa where maize samples were collected.

Studies were conducted in three South African provinces, namely Free State, North-West and Gauteng, which account for over 70 % of the maize produced commercially [18]. These three provinces were further divided into six distinct agro-climatic regions as shown in Fig. 1, namely, WFS (Western Free State), EFS (Eastern Free State), NFS (Northern Free State), SFS (Southern Free State), NW (North West), and GP (Gauteng Province) based on significant differences in maximum rainfall recorded across these agro-climatic regions (Table 1). Samples from these regions were collected from silos. Free State province lies at latitude 28.4541˚ S and longitude 26.7968˚ E; North West province lies at latitude 26.6639˚ S and longitude 25.2838˚ E; and Gauteng province lies at latitude 26.1614˚ S and longitude 28.6442˚ E.

**Table 1:** Mean temperatures and rainfall per agro-climatic region over the years.

**Variables**	**Year**	**Min. Temp. (℃)**	**Max. Temp. (℃)**	**Average Temp. (℃)**	**Min. rainfall (mm)**	**Max. rainfall (mm)**	**Average rainfall (mm)**
**WFS**	2017	14.1	30.7	22.4	1.91	29.59	15.75
	2018	14.68	33.05	23.87	0.74	16.12	8.43
	2019	15.60	31.19	23.40	0.88	21.35	11.12
	2020	14.99	30.45	22.72	3.54	37.95	20.75
	2021	13.64	28.90	21.27	6.90	81.20	44.05
**EFS**	2017	14.34	29.88	22.11	1.88	27.00	14.44
	2018	14.35	32.00	23.18	1.90	44.33	23.12
	2019	15.19	30.00	22.60	2.1	27.33	14.72
	2020	14.94	31.42	23.18	1.96	36.61	19.29
	2021	13.29	27.90	20.60	4.62	43.33	23.98
**NFS**	2017	13.83	30.65	22.24	3.73	46.5	25.12
	2018	14.59	32.61	23.60	1.79	24.46	13.13
	2019	15.20	29.96	22.58	1.79	27.20	14.50
	2020	14.99	30.45	22.72	4.06	42.40	23.23
	2021	13.29	28.40	20.85	3.50	25.87	14.69
**SFS**	2017	12.44	30.84	21.64	1.30	22.00	11.65
	2018	12.90	32.39	22.65	0.40	7.00	3.70
	2019	13.4	30.92	22.16	1.70	45.20	23.45
	2020	12.90	32.39	22.65	0.40	7.00	3.70
	2021	10.93	27.88	19.41	3.12	25.16	14.14
**NW**	2017	13.87	29.55	21.71	2.35	23.00	12.68
	2018	14.84	29.98	22.41	1.80	14.20	8.00
	2019	15.69	31.20	23.45	6.20	98.40	52.30
	2020	14.01	30.45	22.23	2.75	57.60	30.18
	2021	14.19	34.45	24.32	4.05	27.30	15.68
**GP**	2017	14.21	30.33	22.27	1.73	23.20	12.47
	2018	16.16	33.17	24.67	1.00	17.40	9.20
	2019	15.91	29.11	22.51	4.90	64.33	34.62
	2020	16.05	28.84	22.45	3.77	45.30	24.54
	2021	15.66	30.18	22.92	2.79	39.00	20.90

WFS = Western Free State, EFS = Eastern Free State, NFS = Northern Free State, SFS = Southern Free State, NW = North West, GP = Gauteng Province.

### 2.2 Sample collection and preparation

One thousand and twenty-eight (1028) maize samples were randomly collected from selected silos from six agro-climatic regions of SA between 2017 and 2021 lying between the North-West, Free State and Gauteng provinces. Two hundred and fifty (250) maize samples were collected in 2017, 284 samples in 2018, 218 in 2019, 151 in 2020, and 125 in the year 2021. Maize harvested after a short period of drying in the farms by the farmers were brought to the silo for storage. Sample collection was done at this point using the structured sampling model, 10 kg of maize were taken from different points in the silos while the maize was being routinely moved and subsequently mixed, and five kilograms from each of the sampled 10 kg maize were then collected. Then 1 kg of maize was milled to a fine texture with the aid of a milling machine (Retsch, Model ZM200, Germany), placed in sterile zip-lock polythene bags, labelled, and stored at 4°C prior to analysis to halt any further mycotoxin production.

### 2.3 Extraction of Aflatoxins

Aflatoxins were extracted from the milled maize samples by using the Easi-Extract aflatoxin immunoaffinity columns (R-Biopharm Rhone Limited, Glasglow G20OXA, Scotland). Extraction was carried out according to the manufacturer’s protocol, but with some modifications [19]. Two and a half grams of sodium chloride (NaCl) was well mixed into a beaker containing a 250 g maize sample, with 100 ml of an 80 % solution of methanol (methanol:water, 80:20) added to the mixture, which was then well blended for five minutes in an overhead blender. The mixture was filtered through a fluted filter paper (Whatman No.1) into a clean vessel. A volume of 2 ml of the filtrate was then diluted with 14 ml of phosphate buffer saline (PBS) solution and passed through an immunoaffinity column (IAC). The column was washed with 20 ml of PBS and the aflatoxins finally eluted with 1 ml of pure methanol (100 %) into a glass cuvette and stored at −20 °C prior to analysis.

### 2.4 Preparation of calibration standards and validation of the HPLC method

Aflatoxins B_1_, B_2_, G_1,_ and G_2_ standards (Trilogy Analytical Laboratory, Washington, USA) were diluted using acetonitrile (LiChrosolv®, Merk, Germany). The method validation was done by determining parameters highlighted by Adetunji et al. Adetunji et al., [19], namely, linearity, accuracy, and sensitivity. Linearity was determined by constructing calibration curves from standards of AFB_1_, AFB_2_, AFG_2_, AFG_2_, total aflatoxin (AF_tot_) concentration levels by applying linear function functions to the calibration curves. Limit of detection (LOD) and limit of quantification (LOQ) were used to evaluate sensitivity of the method. These were estimated for a signal-to-noise ratio (S/N) × 3 for LOD and (S/N) × 10, for LOQ, from chromatograms of samples spiked before extraction at three additional levels of 25, 50, and 100 ppb for all aflatoxins analysed i.e. AFB_1_, AFB_2_, AFG_2_, AFG_2_, and AFB_Total_^.^ Apart from the 3 concentration levels for each aflatoxin, maize samples of known aflatoxin concentrations were used to determine the linearity of the curve [19]. The LOD was 0.012 ppb whereas LOQ was 0.16 ppb for the machine and an 85 % recovery of the mycotoxins by the extraction method. Regression coefficients (r2) for AFG_1_ was 0.99 whereas for the other three aflatoxins was 1.0 showing good linearity.

### 2.5 High performance liquid chromatography

The HPLC (Shimadzu FCV-20H2; Kyoto, Japan) equipped with a fluorescence detector (a coring cell (CoBrA cell) (Dr Weber Consulting, Mannheim, Germany) was used in this study for the detection and quantification of aflatoxins in maize. The visible derivatization of aflatoxins by the detector was set at 362 nm wavelength for excitation and emission wavelengths were 425 nm (AFB_1_ and B_2_) and 455 nm (AFG_1_ and G_2_). The HPLC analytical column was equipped with a C18 security guard cartridge (Phenomenex, Torrance, CA, USA) and set at 30 ℃ [20]. The mobile phase was modified from the recommended water: methanol ratio (60:40) to a working condition of ratio 55:45, with 119 mg/litre of potassium bromide (KBr) and 1 ml/L of 65 % nitric acid added at a flow rate of 1.0 ml/minute. The volume of injection was 100 μl, and the elution of the aflatoxins was in the order (AF) G_2_, G_1_, B_2,_ and B_1_. The peak areas and retention times of the mycotoxins were used to determine the specific concentration of mycotoxins per sample that was based on those of the standard mycotoxins determined from using the calibration curve.

### 2.6 Climate information

Climatic data (daily rainfall and temperature) from 2017 to 2021 were collected from the South African Weather Services (SAWS) from several districts situated in the Free State, North-West and Gauteng provinces. The lowest minimum mean temperature (10.93 ℃) was recorded in South Free State in 2021, while the highest maximum mean temperature (33.05 ℃) was recorded in Western Free State in 2018. In 2018, the lowest mean minimum rainfall (0.40 mm) was recorded in SFS, while the highest maximum (98.40 mm) was recorded in NW in 2019. Table 1 shows the detailed climatic information (Maximum and minimum temperatures and rainfall) of the locations over the study period.

### 2.7 Statistical analyses

The general linear models (Proc Glm) of SAS (2010) were used to analyse the correlations between AF level, year and agro-climatic region, calculated by the Pearson Correlation (rho), using the PROC CORR procedure of SAS (2010).

## 3 Results

### 3.1 Rate of aflatoxin contamination of maize

**Fig. 2:**
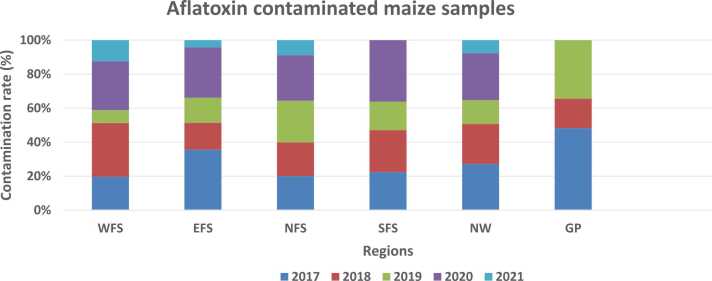
Contamination rate of maize with aflatoxin.

A total of 1028 maize samples were collected from different agro-climatic regions and analysed in this study. Two hundred and five (19.94 %) maize samples were contaminated with aflatoxins. While samples collected during the farming season of 2021 had the lowest contamination rate (8 %), the year 2020 presented the highest contamination rate (25.83 %) (As presented in Fig. 2).

### 3.2 Summary of regional means for aflatoxin contamination per annum and per agro-climatic region

**Fig. 3:**
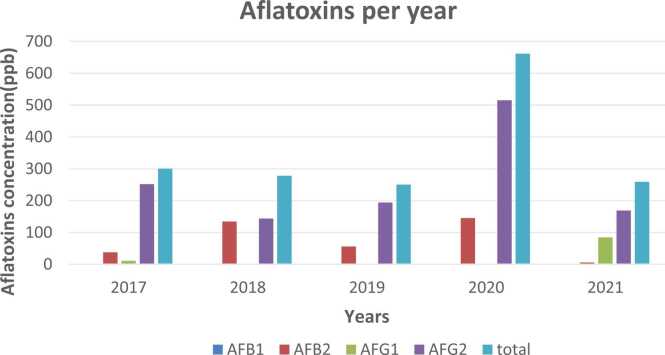
Annual mean aflatoxin contamination.

In all, the 2020 harvest season recorded the highest mean values of the different aflatoxins, while the 2021 harvest season presented with most of the lowest values. In all, the mean total aflatoxin concentration level amongst positive samples in this study was 64.17 ppb. Fig. 3 presents the different mean aflatoxin concentrations.

**Fig. 4:**
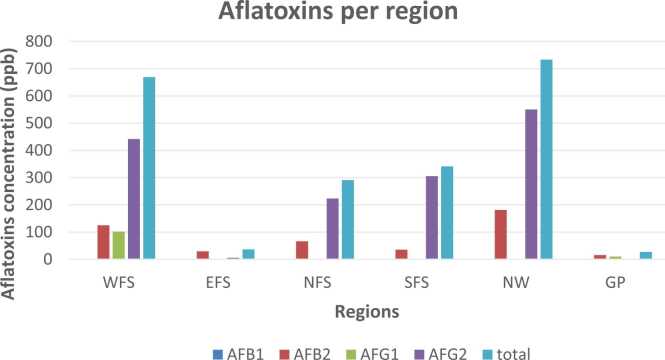
Regional mean aflatoxin contamination.

In all, NW and WFS recorded most of the highest aflatoxin mean concentration, while GP and EFS the least aflatoxin mean values (Fig. 4).

It has been established in this study that the overall mean total aflatoxin concentration level amongst all positive samples in SA commercial maize is 64.17 ppb, and that none of the samples were contaminated with AFB_1_ above the SA regulatory limit of 5 ppb. Table 2, Table 3 further presents in details percentages of samples exceeding the SA limits of mean total aflatoxin concentration for human (10 ppb) and animal consumption (20 ppb) per year in different agro-climatic regions. Out of the 19.94 % of maize samples contaminated with aflatoxins, 64.39 % had mean total aflatoxins above SA regulatory limit (Table 2). In 2018 for instance, all maize samples contaminated with aflatoxins (100 %) had mean total aflatoxin above SA set limit in maize destined for human consumption, and 98.55 % above SA set limit in maize destined for animal consumption (Table 2). While NFS had the most samples contaminated with mean total aflatoxins above SA set limit for maize destined for both human and animal consumption of 82.35 % and 70.59 % respectively, EFS was the least (33.33 %) (Table 3).

**Table 2:** Percent of samples exceeding the SA limits of mean total aflatoxins per annum.

**Year**	**Positive samples**	**Positive samples above 10 ppb**		**Positive samples above20 ppb**	
		**n**	**%**	**n**	**%**
**2017**	58	10	17.24	5	8.62
**2018**	69	69	100	68	98.55
**2019**	29	14	48.28	10	34.48
**2020**	39	37	94.87	36	92.31
**2021**	10	2	20	2	20
**Total**	**205**	**132**	**64.39**	**121**	**59.02**

**Table 3:** Percent of samples exceeding the SA limits of mean total aflatoxins per agro-climatic region.

**Agro-climatic Regions**	**Positive samples (n)**	**Positive samples above 10 ppb**		**Positive samples above 20 ppb**	
		**n**	**%**	**n**	**%**
WFS	77	49	63.64	47	61.04
EFS	18	6	33.33	4	22.22
NFS	34	28	82.35	24	70.59
SFS	27	17	62.96	17	62.96
NW	42	29	64.44	26	61.90
GP	7	3	42.86	3	42.86
**Total**	**205**	**132**	**64.39**	**121**	**59.02**

## 4 Discussion

South Africa is a leader in Africa in terms of maize production and exportation. With the socio-economic and health effects associated with aflatoxins, it has become a global issue especially in the face of climate change, and effective mitigatory methods are needed. Therefore, this study set out to examine aflatoxin contamination of South African commercial maize cultivated in different agro-climatic regions. About 20 % (19.94 %) of South African commercial maize samples were found to be contaminated with aflatoxins. The mean total aflatoxin concentration level amongst all positive maize samples in this study was 64.17 ppb, which is far above the prescribed regulatory limit of 20 ppb set by South Africa. Different mean values for total aflatoxins were observed for different years as well as different agro-climatic regions and this disparity can be attributed to climate variability. In fact, South Africa has been experiencing frequent droughts, and a good number of studies have associated high levels of aflatoxins in crops with the occurrence of drought [13], [21]. In this study, mean total aflatoxin concentration levels was found to be significantly associated with agro-climatic regions and the year in which the maize was harvested except for SFS. In the light of the analysis of the weather conditions from these regions and the relevant years covered by this research study, it was evident that the overall average rainfall from SFS was very low as opposed to that of the other agro-climatic regions (Table 1). Furthermore, the rise in annual temperature (32±1.5 ℃) and the corresponding decline in annual rainfall in the years 2018 and 2020 in WFS, NFS and NW resulted in a significant rise in total aflatoxin production which corroborate other studies [9], [21], [22]. The findings of this research (32±1.5 ℃, in addition to drastic reduction of rainfall in particular) corroborate those of Paterson and Lima [23] who reported warmer temperatures of 33 °C to be optimal for aflatoxin production in maize and also other studies that reported maximum aflatoxin production at 28 °C Gallo et al. [24] and 30°C Garcia-Cela et al. [25]. The locations where maize samples used in this study lie within latitudes 26.1416 and 28.4541°S which has reported to register the highest levels of mycotoxin contamination in crops Atukwase et al. [26].

In SA, regulatory limits exist for AFB1 (5 ppb) and total aflatoxins (10 ppb) in food and for total aflatoxins in feed (20 ppb). In this study, throughout the years and in all agro-climatic regions, AFB_1_ contaminated about 15 % (14.87 %) of the overall samples (1028) of which none had mean value above the SA set limit of 5ppb. While out of the contaminated samples (205 samples or 19.94 %), 64.39 % were contaminated with mean value of AF_Tot_ above the SA set regulatory limit (10 ppb) for human consumption and 59.02 % samples contaminated with mean value of AF_Tot_ above the SA set regulatory limit (20 ppb) for animal consumption. To a certain degree, it might be safe to say the most carcinogenic aflatoxin (AFB_1_) is present within regulatory limits in SA commercial maize, but on a second thought, the heavy contamination of maize by the other types of aflatoxins above 10 ppb and 20 ppb for both human and animal consumption respectively cannot be completely ignored. With a mean total of aflatoxins of 64.17 ppb in this study, all the years except for 2021 with acceptable levels of mean total aflatoxins contamination fit for human consumption (2.54 ppb), which corroborates earlier study by [13], with mean total aflatoxins of 2.40 ppb in SA commercial maize harvested in 2015/2016 harvest season. All the other years had high mean values (2017 and 2019) and extremely high mean values (2018 and 2020) above both the set limit of animal and human consumption. For instance, in 2018, all maize samples contaminated had aflatoxins above the set regulatory limit. Furthermore, while GP agro-climatic region mean total aflatoxin was fit for animal consumption (19.19), it was above set limit for human consumption. All the other regions had two (EFS, SFS and NFS) to five folds (NW and WFS) the regulatory limit for animal consumption. Hence, if care is not taken, with rising temperatures and sporadic rainfall pre-harvest aflatoxin contamination risk of SA commercial maize will be of safety concern sooner than later.

## 5 Conclusions

Almost twenty percent (19.94 %) of SA commercial maize samples are contaminated by aflatoxins, with a mean total aflatoxin concentration level of 64.17 ppb amongst the contaminated samples, which is above the SA regulatory limit of 20 ppb set for animal consumption. Amongst the contaminated samples, 59.02 % had mean total aflatoxin concentration levels above the SA regulatory limit of 20 ppb for animal consumption. No sample contaminated by the regulated aflatoxin B_1_ class was above the prescribed SA level of 5 ppb. Climate variables significantly influence the aflatoxin contamination of South African maize (especially a decline in the average annual rainfall led to increased levels of aflatoxin contamination of maize.

## Funding

This research, together with the APC, was funded by North West University, Mafikeng.

## CRediT authorship contribution statement

**Queenta Ngum Nji:** Writing – review & editing, Writing – original draft, Methodology, Formal analysis, Data curation. **Olubukola Oluranti Babalola:** Writing – review & editing, Visualization, Validation, Supervision. **Mulunda Mwanza:** Validation, Supervision, Resources, Project administration, Funding acquisition, Conceptualization.

## Declaration of Competing Interest

The authors declare the following financial interests/personal relationships which may be considered as potential competing interests: None reports was provided by None. None reports a relationship with None that includes:. None has patent Not applicable pending to Not applicable. Not in this case If there are other authors, they declare that they have no known competing financial interests or personal relationships that could have appeared to influence the work reported in this paper.

## Data Availability

Data will be made available on request.
