# Supplementary material for: Climatic effects on aflatoxin contamination of maize
Source: Toxicol Rep. 2024 Aug 15;13:101711. doi: 10.1016/j.toxrep.2024.101711 (PMC11388663; doi:10.1016/j.toxrep.2024.101711)
Supplement: Supplementary file 1 — Supplementary material [file mmc1.docx]

**Supplementary Materials**

Supplementary table 1: Frequency of contamination for the different aflatoxin types per annum.

| **Year** | **Afflatoxin contamination** | | | | | | | | | |
| --- | --- | --- | --- | --- | --- | --- | --- | --- | --- | --- |
|  | **Total** | | **G_2_** | | **G_1_** | | **B_2_** | | **B_1_** | |
|  | **Frequency** | **Rate (%)** | **Frequency** | **Rate (%)** | **Frequency** | **Rate (%)** | **Frequency** | **Rate (%)** | **Frequency** | **Rate (%)** |
| **2017** | 58/250 | 23.2 | 15/58 | 25.86 | 47/58 | 81.03 | 21/58 | 36.21 | <LOD | <LOD |
| **2018** | 69/284 | 24.3 | 23/69 | 33.33 | <LOD | <LOD | 69/69 | 100 | <LOD | <LOD |
| **2019** | 29/218 | 13.3 | 27/29 | 93.1 | 2/29 | 6.7 | 19/29 | 65.52 | 9/29 | 31.03 |
| **2020** | 39/151 | 25.83 | 21/39 | 53.85 | 3/39 | 7.69 | 37/39 | 94.87 | 13/39 | 33.33 |
| **2021** | 10/125 | 8 | 9/10 | 90 | 4/10 | 40 | 5/10 | 50 | 1/10 | 10 |

Supplementary table 2: Frequency of contamination for the different aflatoxin types per agro-climatic region.

| **Region** | **Afflatoxin contamination** | | | | | | | | | |
| --- | --- | --- | --- | --- | --- | --- | --- | --- | --- | --- |
|  | **Total** | | **G_2_** | | **G_1_** | | **B_2_** | | **B_1_** | |
|  | **Frequency** | **Rate (%)** | **Frequency** | **Rate (%)** | **Frequency** | **Rate (%)** | **Frequency** | **Rate (%)** | **Frequency** | **Rate (%)** |
| **WFS** | 84/423 | 19.86 | 28/423 | 6.62 | 26/423 | 5.15 | 60/423 | 14.18 | 6/423 | 1.15 |
| **EFS** | 17/56 | 30.36 | 8/56 | 14.29 | 7/56 | 12.5 | 14/56 | 25 | 2/56 | 3.57 |
| **NFS** | 33/184 | 17.93 | 17/184 | 9.24 | 8/184 | 4.35 | 26/184 | 14.13 | 1/184 | 0.54 |
| **SFS** | 25/98 | 25.51 | 15/98 | 15.31 | 2/98 | 2.04 | 18/98 | 18.37 | 5/98 | 5.1 |
| **NW** | 44/235 | 18.72 | 26/235 | 11.06 | 11/235 | 4.68 | 34/235 | 14.47 | 6/235 | 2.55 |
| **GP** | 7/32 | 21.88 | 3/32 | 9.38 | 3/32 | 9.38 | 3/32 | 9.38 | 1/32 | 3.13 |

**Aflatoxin calibration curves**

Standard curves were constructed by calculating the ratio of the peak areas for each aflatoxin standard sample spiked before and after extraction at three additional levels of 25, 50, and 100 ppb for all aflatoxins analyzed (AFB_1_, AFB_2_, AF_tot_) (Supplementary figures 1 a, b c, and d). The toxins (G_2_, G_1,_ B_2,_ and B_1_) were eluted at 6–7, 7–8, 8–9, and 10–11 minutes respectively. The aflatoxin content of the maize samples was measured in ppb.

1. b)

1. d)

Supplementary figures 1 a-d: Standard curves highlighting the lighting the linear regression of HPLC method for AFG_2_, AFG_1_, AFB_2_, and AFB_1_, respectively.

Supplementary Table 3: Different aflatoxins and their mean concentrations for the respective years and agro-climatic regions.

| **Year/Agro-climatic region** | **Types of aflatoxins and their mean concentration (ppb)** | | | | | | | | | | | | | | | | | | | |
| --- | --- | --- | --- | --- | --- | --- | --- | --- | --- | --- | --- | --- | --- | --- | --- | --- | --- | --- | --- | --- |
|  | **2017** | | | | **2018** | | | | **2019** | | | | **2020** | | | | **2021** | | | |
|  | **B_1_** | **B_2_** | **G_1_** | **G_2_** | **B_1_** | **B_2_** | **G_1_** | **G_2_** | **B_1_** | **B_2_** | **G_1_** | **G_2_** | **B_1_** | **B_2_** | **G_1_** | **G_2_** | **B_1_** | **B_2_** | **G_1_** | **G_2_** |
| WFS | 0 | 80.23 | 4.16 | 679.98 | 0 | 165.63 | 0 | 509.38 | 0.21 | 81.3 | 0 | 3.14 | 0 | 289.69 | 0 | 1015.1 | 0.1 | 9.67 | 505.8 | 1.58 |
| EFS | 0 | 4.2 | 4.89 | 20.17 | 0 | 137.58 | 0 | 9 | 0 | 0 | 0 | 0 | 0 | 0 | 0 | 0 | 0 | 5.9 | 0.5 | 0 |
| NFS | 0 | 11.74 | 0.85 | 0 | 0 | 106.15 | 0.53 | 337.57 | 0.1 | 24.41 | 1.3 | 7.22 | 0.5 | 189.96 | 3.2 | 771.4 | 0 | 0 | 0 | 0.62 |
| SFS | 0 | 0 | 2.41 | 0.78 | 0 | 138.21 | 0 | 2.37 | 0 | 18.15 | 0 | 338.98 | 0.27 | 22 | 0 | 1184.8 | 0 | 0 | 0 | 0 |
| NW | 0 | 123.7 | 3.44 | 807.7 | 0 | 203.6 | 0 | 4.83 | 0 | 190.9 | 0 | 809.43 | 0.44 | 369.03 | 4.5 | 119.6 | 0 | 18.5 | 0 | 1010.9 |
| GP | 0 | 5.25 | 50.5 | 1.68 | 0 | 53.78 | 0 | 0 | 0.1 | 21 | 0.6 | 4.32 | 0 | 0 | 0 | 0 | 0 | 0 | 0 | 0 |
